# Artificial Intelligence (AI) and Lung Ultrasound in Infectious Pulmonary Disease

**DOI:** 10.3389/fmed.2021.706794

**Published:** 2021-11-25

**Authors:** Guglielmo Trovato, Matteo Russo

**Affiliations:** The European Medical Association (EMA), Brussels, Belgium

**Keywords:** artificial intelligence, deep learning-artificial neural network (DL-ANN), ethics, lung imaging, bullying in international settings

## Summary

Deep Learning algorithms are ready for use and can aid in the interpretation of radiological findings monitoring change during follow-up. However, the translational approach to ultrasound is difficult and currently subject to major limitations. Great help can derive from computer-assisted report procedures, which, in appropriate contexts of expert machine learning, can structure a future context of diagnostic orientation.

Lung ultrasound is a diagnostic method that relies heavily on the operator's abilities, in which the human expertise component must be accompanied by the most suitable quality of the equipment and of its appropriate setting. However, this strictly clinical and qualitative interpretation when reading lung images is an element of weakness since we have health professionals with different levels of knowledge and expertise in performing the procedure. There are also particular difficulties linked to the conditions of the patients and to the methods of assistance and caution due to the need to prevent contagion in the fight against coronavirus 2019 disease (COVID-19).

Patients, particularly in limited resource subsets at home, in ambulances, and in emergency room facilities, are very difficult subjects even for expert operators, and this is also because chest ultrasounds are full-contact procedures.

Quantitative analysis makes radiology reporting much more comprehensive. Actually, several research groups have begun looking to artificial intelligence (AI) as a tool for reading and analyzing X-rays and computed tomography (CT) scans and helping to diagnose and monitor COVID-19 by several deep-learning approaches. This is carried out with the hope of overcoming the intrinsic limits of the diagnostic procedure and the context of intervention.

Unfortunately, with US procedures, the proposal to use inappropriate methods, based on the automatic reading of the artifacts, in particular of the b-lines, has been addressed: this is what has been unexpectedly, and for no good reason, suggested by many. In this regard, it must be strongly reiterated that quantifying erratic and unreliable artifacts, measuring them by machine counting methods, is only a mystifying and misleading approach without advantages and is de facto dangerous in patient management.

Currently, it is unlikely that the algorithms proposed may directly replace the medical doctors, and, namely, the radiologists' judgments as well as personal responsibility during ultrasound diagnosis. This is due to the limited or even absent specificity of these approaches for categorizing definite findings and to the medico-legal implications of such diagnoses.

## Overview

Research, development, application, and dissemination of Artificial Intelligence (AI) in imaging and vision techniques can allow us to gather a great deal of complex information as well as provide very direct diagnoses from available data. Current computer vision techniques powered by deep learning for medical applications have their focus on medical imaging, medical videos, and clinical deployment (1). A still unresolved challenge is that clinical workflows, encompassing different sources and features of data, should integrate computer vision to enhance medical practice and health care. Currently, several research groups have begun looking to artificial intelligence (AI) as a tool for reading and analyzing X-rays and computed tomography (CT) scans, helping to diagnose and monitor COVID-19 by several deep-learning approaches. Lung ultrasound is a diagnostic method greatly operator-dependent, in which the human expertise component must be accompanied by the most suitable quality of the equipment and its appropriate setting (2). As a consequence, this strictly clinical and qualitative interpretation reading of lung images is an element of weakness when health professionals with different levels of knowledge and expertise in performing the procedure are operating (3). Patients, particularly in limited resource subsets at home, in ambulances, and in emergency room facilities, are very difficult subjects for a full-contact procedure such as the chest ultrasound. Specific difficulties are related to the clinical conditions of the patients and to the methods of assistance and caution due to the need to prevent the spread of infection in the fight against coronavirus disease 2019 (COVID-19). This makes it even more difficult to perform the exam (4, 5). The extent of the lung ultrasound (LUS) was an important factor in the diagnosis of COVID-19 pulmonary involvement, and it is still widely debated today, especially with regard to the differential diagnosis between different pathologies (6). Indeed, the point is that chest ultrasound does result in false negatives in most cases, even when performed with a great level of expertise and scrutiny (6). Overall, Lung Ultrasound (LUS) should not be considered as a reliable imaging tool in ruling out COVID-19 pneumonia in any patient, in Emergency or elective subsets. This is demonstrated according to unbiased experience and accurate assessment of actual achievements (6). The US method of lung imaging, especially in the diagnosis of pneumonia, whatever its cause, including COVID-19, finds its application role in the integration of other more sensitive and specific diagnostic tests, such as the nasopharyngeal swab, virus molecular assays, and Chest-CTs.

## Crossing and Overlapping

Are there any cross points or overlaps between radiological profile imaging, namely, plain chest X-rays (CXR), Computerized Tomography (CT), Nuclear Magnetic Resonance NMR, and Ultra-Sound (US) imaging? Yes, of course. Nonetheless, a brief discussion on how it has been possible to progress with deep learning approaches in the fields of AI vision machinery may still be useful. Medical images have peculiar characteristics, nowadays often already coming from digital signal processing. For these reasons, they represent a series of challenges to machine vision based on deep learning (1). Computer vision in radiology has a greater focus on chest X-ray analysis, a field that has collected millions of annotated, open-source images (7). Such approaches should have enabled the field to respond rapidly in times of crisis, for instance, developing and deploying COVID-19 detection models (8). This area of research and application continues to expand with work in image translation (e.g., striving to convert noisy ultrasound images into Magnetic Resonance Imaging) (1) and with other advances in computational lung ultrasound imaging (9).

Image processing methods may be split into two categories: (1) traditional model-based methods and (2) data-driven methods. For the former, inverse problems are based on methods focusing in particular on ultrasound imaged speckling, deconvolution, and line artifacts detection. For the second method, among the data-driven approaches, various machines, as well as deep learning procedures, have been applied in the contexts of supervised, weakly supervised, and unsupervised learning. These include the implementation of various effective network architectures, in the case of deep learning, and high-dimensional statistical methods in the case of the more traditional machine learning (9). Nonetheless, given that ultrasound images involve operator-, patient-, and scanner-dependent variations, the adaptation of classical machine learning methods to clinical applications becomes challenging, and such challenges still constitute a major limitation in Lung US imaging, even without any automation of interpretation, more if reasonably and unpredictably amplified by digital approaches based on uncertain, erratic and misleading data (10). Differently, in radiology, a deep learning model, the COVID-19 detection neural network (COVNet), was developed to extract visual features from volumetric chest CT scans for the detection of COVID-19. CT scans of community-acquired pneumonia (CAP) and other on-pneumonia abnormalities were included to test the robustness of the model. The conclusion is that such a model accurately detects coronavirus 2019 pulmonary disease and differentiates it from community-acquired pneumonia and other lung conditions (11). Using CX-Rays, even if deep neural networks models are suitable to classify most chest X-ray images into a specified target class, careful consideration is required. Their practical applications to COVID-19 diagnosis are not yet sufficiently safe due to the need for strategies to address security concerns (12). This warning is mostly relevant considering several overenthusiastic reports, claiming a very successful but unlikely application of such methods (13, 14).

## Appropriateness and Reliability—Mistaken Proposals

Regarding the appropriateness of the use of automatic digital analyzes in thoracic ultrasound, all the claims of great success have been regularly denied in the medical journals that have published timely articles and comments (15–17). In many of our previous detailed comments, the appropriateness of methods based on ultrasound artifacts, that is, on the operator's inability to obtain visible images, was denied. Ultimately, it is like saying that if an inexperienced operator is unable to perform an adequate examination with meaningful images, getting only erratic artifacts, then the pathology is present (18). As a consequence, and certainly much worse, some groups tried to accredit the concept that at greater levels of difficulty in acquiring meaningful images, there are different or more severe pathologies (19). Finally, an attempt has been made to give these approaches, based on the measurement of information lacking any reliability or reproducibility, the role, and rank of objective automated measurements (20, 21). All this has reached the level of unofficial recommendations. Such “guidelines,” under so great inconsistency, can only be considered as mistaken proposals, particularly dangerous when they are aimed at replacing the doctor's action in carrying out the examination, substituting their clinical judgment for a diagnostic evaluation, even among COVID-19 pandemics and emergency referrals (22–24).

## Clinical Unmet Needs and the Marketplace Reasons

At this point, an analysis of this situation is necessary. What are the unmet needs? Of these, there are too many. The focus is on the search for the advantages of performing an examination on the patient without irradiation risk, being safe with regard to the possibility of inter-human contagion, being performable at the patient's bedside (or in any case in the treatment area), being reproducible in any context and by any operator, and being appropriate for use as a preferential diagnosis with respect to radiological methods. The answer is, unfortunately, that these needs are not met at all either by the thoracic ultrasound that uses images, but still less, by approaches based on the empiricism of erratic tests counting and searching for b-lines, i.e., artifacts due to bad machine settings, unskilled operators, difficult or impossible echographic window, or a melting pot of all concurrent biases (25).

The measurement of chaotic artifacts, which is very dependent on factors not related to the specificity of the pathologies, must be acknowledged as not being the desired solution (26). In an era of managed healthcare, the reasons, interventions, and conflicts present in the marketplace must be taken into consideration (1). However, apart from advertising methods and tools that are claimed to provide answers of excellent diagnostic quality, there is nothing that suggests which improvement in managed health care might currently ensue from a generalized deep learning approach. This should be developed (and has not yet been done) within transparent clinical trials focused on risk analysis and management mitigation plans. Only this will allow avoiding incorrect or delayed diagnosis, which is in fact the only certainty we can hold on to on inconsistent bases, as are currently and sparsely proposed. Optimal approaches, in our view, should be targeted to the training and quality enhancement of single operator skills, knowledge, and interpretation (27).

## Conclusion

The proposal to use inappropriate methods, based on the automatic reading of artifacts, namely, of B-lines, has returned unexpectedly, and for no good reason, from many sides, and must be reasonably rejected. In this regard, it must be strongly reiterated that quantifying erratic and unreliable measurements by machine counting methods is only a mystifying and misleading approach, without advantages, and is de facto dangerous in patient management. These beliefs are detrimental for the other best-supported ultrasound practices in any subset, including emergency and pediatrics (28, 29). Lung ultrasound allows detecting lung consolidations in many patients with CXR-confirmed Community-Acquired Pneumonia, even giving, regretfully, false-negative results in a quarter of cases. Previous longitudinal results confirm its role in the follow-up of detectable lesions. Nonetheless, ultrasound should be regarded only as a complementary and monitoring tool in pneumonia, instead of a primary imaging modality (30).

The claims of lung ultrasound usefulness in COVID-19 pneumonia are unsupported. Namely, it should not be considered an alternative to CT scan for assessment of COVID-19 pneumonia in pregnant women, especially considering the higher risk of operator exposure to contagion associated with this type of examination (31). Moreover, Lung US findings in COVID-19 patients are even less specific than those detectable on CT scans are (32).

Deep Learning algorithms can aid in the interpretation of radiological findings and may help for monitoring their change over follow-up. However, the translational approach to ultrasound is intrinsically difficult, lacks any evidence basis, and is currently still subject to major limitations. Great help can be derived from computer-assisted report procedures, which, in appropriate contexts of expert machine learning, can structure a future more harmonic context of shared diagnostic orientation. Currently, it is unlikely that the algorithms proposed may directly replace the medical doctors', and, namely, the radiologist's, judgment and responsibility in ultrasound diagnoses. This is due to the limited or absent specificity of these approaches for categorizing specific findings and to the medico-legal implications of such diagnosis. Requirements regarding certifiability of AI approaches and applications are of utmost relevance (Table 1) and far from being currently achieved in Lung US imaging.

**Table 1:** Requirements for a suitable use of deep learning in Lung imaging: what the lung ultrasound still lacks. Key messages.

**Interpretability**	**Guarantees**	**Certifiability**
Due to the over-parameterized nature of Deep neural network (DNN) models, they are usually treated as black-boxes and their predictions never questioned. Especially in medicine, where wrong predictions may have disastrous consequences, interpretability of results is of the uttermost importance. This may mean that the model output is not only the bare prediction but also what feature and quality of the input data caused such prediction. This may ensure that a medical operator can always interpret, explain and challenge DNNs predictions	For especially high-dimensional inputs and extremely over-parameterized network architectures, explainability in the form of interpretability may be computationally hard to achieve and, thus, impractical. Nonetheless, there exist already statistical frameworks that can be juxtaposed to the DNN and give out guarantees on the output distribution	When Deep Learning systems are sold to hospitals, they have to come with a certification of the output prediction in order to ensure not only statistical consistency but also serve as a basis for legal compliance

## Author Contributions

All authors listed have made a substantial, direct and intellectual contribution to the work, and approved it for publication.

## Conflict of Interest

The authors declare that the research was conducted in the absence of any commercial or financial relationships that could be construed as a potential conflict of interest.

## Publisher's Note

All claims expressed in this article are solely those of the authors and do not necessarily represent those of their affiliated organizations, or those of the publisher, the editors and the reviewers. Any product that may be evaluated in this article, or claim that may be made by its manufacturer, is not guaranteed or endorsed by the publisher.
